# From Waste to Value: Urine and Ash as Sustainable Sources for Green Ammonia and Calcium Phosphate Fertilizers

**DOI:** 10.3390/bioengineering13070720

**Published:** 2026-06-24

**Authors:** Zhengyu Li, Eduard Tiganescu, Kevin Böhm, Muhammad Jawad Nasim, Claus Jacob

**Affiliations:** Division of Bioorganic Chemistry, School of Pharmacy, Saarland University, 66123 Saarbruecken, Germany; zhli00012@stud.uni-saarland.de (Z.L.); s9edtiga@stud.uni-saarland.de (E.T.); kevin.boehm@uni-saarland.de (K.B.)

**Keywords:** ash, ammonia, circular (bio)economy, green, phosphate, *Providencia rettgeri*, struvite, urine, waste-to-value

## Abstract

Turning waste materials into renewed value is a central aspect of any future circular (bio)economy. Urine and ash are two prominent waste materials produced globally and in considerable amounts each year. Both contain several interesting substances that are so far de facto lost or may even pose a threat to the environment. Urine from industrial-scale farming, for instance, is responsible for significant pollution of soil and groundwater with nitrogen and phosphorous, yet N and P are also high-demand substances in agriculture and industry. Similarly, ash is rich in several interesting metal ions, but is still usually disposed of in a landfill. Using a sequence of simple yet effective biological and chemical processes, it may be possible to convert these two unwanted materials into “green” ammonia and calcium phosphate, both valuable high-demand substances with numerous applications, and with few potentially valuable leftovers still to be dealt with. Eventually, and after considering some of the logistics of the process, such as the collection of materials, this “urinash process” may be upscaled to effectively reduce waste by turning it into renewed value, thus also substituting for—some of—both the energy-intensive synthesis of grey ammonia and the destructive mining for phosphate salts.

## 1. Introduction

These days, the challenges facing the global economy are manifold. They include, among others, both acquiring a sustainable supply of green fuels and raw materials, such as fertilizers, on the one hand, and increasing amounts of waste on land and in the oceans on the other [1,2,3,4]. The idea of switching from linear material flows to circular ones is therefore highly attractive, at least in theory, as it may simultaneously resolve both of these issues at once [5,6,7,8,9]. In practice, however, the situation is more complex, as waste may not always be in the right place and right hands at the right time [10]. Furthermore, many waste materials are more or less “dirty”, and thus may not easily be converted into valuable materials, as such conversions may require excessive amounts of energy; extraction or purification procedures and themselves may produce additional waste or simply fail because of logistics (e.g., accessibility, collection, transportation, storage, handling of waste materials) [11]. Spent coffee grounds (SCGs), for instance, can be used in shampoos or cosmetic scrub bases, yet going from door to door to collect handfuls of SCGs each day and to subsequently process this material before it rots poses a real challenge, which makes large-scale handling difficult and economically unattractive [12].

Among the more promising waste materials that still mostly go down the drain today are (animal) urine and wood ash. Together, both may be “combined” to provide nitrogen, phosphorous, calcium and magnesium, the basis of many fertilizers currently in high demand. Since these resources may be sourced locally, they could even provide an additional level of economic activity and resilience within existing supply chains, thereby helping to mitigate risks associated with disruptions to major trade routes, such as the Strait of Hormuz. Indeed, dealing with these two apparent waste materials in one go is attractive, as it is estimated that 74 million tons of urine from cattle and one million tons of wood ash are produced each year in Germany alone, and both of these numbers are likely to increase considerably in the near future, not least because of excessive meat consumption on the one hand and the use of wood as a renewable energy source on the other [13,14,15,16,17,18].

In theory, the estimated 74 million t of animal urine could yield approximately 1.1 million t of urea. Although this represents only about 0.55% of current global urea production (201 million t), it could make a significant contribution to domestic waste recycling and fertilizer security within a country such as Germany. In rural regions, it may even provide new opportunities for economic activity and employment.

Urine is especially rich in nitrogen (N) and phosphorous (P), and the 74 million t just mentioned corresponds to about 1.1 million t of urea and 164,400 t of phosphate on average, although it is important to mention that the actual composition of urine is subject to strong fluctuations and may therefore differ in reality from the assumed values [19]. Today, neither nitrogen nor phosphorous is being recovered, and both de facto go to waste. Eventually, this also turns into a major environmental problem as both N and P already exceed the safe environmental boundaries, with dramatic consequences for flora, fauna and also for us humans [20]. To address this environmental issue, human urine is often channelled to sewage treatment plants, where bacteria such as *Nitrosomonas*, *Nitrobacter* or *Thauera* convert its nitrogen to nitrate or N_2_, respectively [21,22,23]. Still, nitrate itself is highly problematic as it may enter the environment and contaminate the groundwater [24]. There are also several other methods currently being explored to recover a few main components of urine. Some of them are summarized in Table 1 and, besides being still experimental, most of them have moderate to high operational requirements and are energy-intensive.

Compared to human urine, animal urine is less efficiently handled and indeed is a major contributor to environmental pollution, as most of the liquid manure from intensive animal farming on an industrial scale is simply discarded, often even illegally. Increasingly, attempts to collect and process manure are being considered, yet the logistics behind collecting and processing manure on an industrial scale are challenging—and few of the players and stakeholders involved in this business like to “talk bullshit” either, literally.

Wood ash is another waste material rich in calcium and other valuable minerals. It is produced annually on a million-ton scale in Germany, for instance, by small, combined heat and/or power stations, which are becoming increasingly popular thanks to their reliance on wood as a—supposedly renewable—fuel. Unlike oil or gas, wood and waste from wood processing industries (such as the furniture industry) are considered sustainable and CO_2_-neutral. Still, such power stations produce considerable amounts of wood ash, which is mostly composed of carbonates (CO_3_^2−^) and oxides [35]. The small 882 kW heat station in Sankt Ingbert, Saarland, Germany, for instance, which produces hot water for five households and 25 companies in a small industrial area, converts 3915 loose cubic meters of wood chips to around 18 t of wood ash each year. Like animal urine, wood ash is often produced in rural areas; i.e., both waste materials occur in close(er) geographic vicinities. Unlike urine, this ash can be collected and handled easily, yet despite being rich in minerals, it also still has no real value and is usually discarded in landfills. The calcium content of up to 18% could be particularly useful, for instance, for the production of cement, gypsum or, indeed, as will be shown in this manuscript, green Ca_3_(PO_4_)_2_.

In order to navigate this fertile strait towards a sustainable supply of green substances further, we now report a sequence of biological and chemical processes that produces new value from both ash and urine and shall be referred to as the “urinash process”. It should be noted from the beginning that this initial feasibility study deals with rather simple materials and thus intentionally relies on an equally simple and robust chemistry and biology, and that optimization of the various processes needs to be addressed in more detail in a follow-on study.

## 2. Materials and Methods

### 2.1. Materials

Chemicals were purchased from commercial sources and used without further purification. Wood ash from wood chips burnt at between 780 °C and 980 °C (with temperatures up to 1200 °C possible in the glow) was kindly provided by Stadtwerke St. Ingbert GmbH (St. Ingbert, Germany).

### 2.2. Preparation of Artificial Urine Samples

Multipurpose artificial urine (MPAU) was prepared according to the literature [36]. The exact composition of MPAU is provided in the Supplementary Information (SI) in Table S1.

### 2.3. Enzymatic Conversion of Urea to Ammonia and Analysis

Initially, commercially available urease (EC 3.5.1.5) was employed to produce NH_3_ from the urea present in MPAU. The enzymatic concentration of urease was optimized, and a concentration of 10 U mL^−1^ was chosen for the enzymatic reactions. A total of 200 units of urease were added to each 20 mL of MPAU, and the reactions were continued for five hours. At each designated timepoint, including timepoint 0, the reaction mixtures were analyzed for the concentration of ammonia and phosphate as well as the pH. The concentration of ammonia was determined using the Berthelot assay according to the protocol described in the literature [37,38]. A calibration curve of NH_4_Cl was constituted, ranging in concentrations from 0 to 25 mM. The concentration of phosphate was quantified using the molybdenum blue assay according to the literature [39,40]. The calibration curve of phosphate was established using disodium hydrogen phosphate in concentrations ranging from 0 to 1 mM. An Epoch 2 microplate reader (BioTek Instruments GmbH, Bad Friedrichshall, Germany) was utilized for both spectroscopic assays. pH values were measured using a calibrated FiveEasy™ pH meter (Mettler Toledo, Greifensee, Switzerland) equipped with a standard glass electrode. All experiments were performed in triplicate and on three different occasions (*n* = 9).

### 2.4. Selection and Identification of Robust Urease-Containing Bacteria

*Providencia rettgeri* was isolated from a household urinal and identified using MALDI-TOF by Prof Markus Bischoff at the Institute of Medical Microbiology and Hygiene, Saarland University, Homburg, Germany. Briefly, 10 µL aliquots of the sample were plated on the following plates: Tryptic Soy Agar (TSA)–sheep blood (non-selective); MacConkey (selective for Gram-negative bacteria); Chocolate agar (needed for growth of fastidious organisms); Columbia Nalidixic Acid (CNA) agar (selective for Gram-positive bacteria); and Schaedler Agar (used for cultivation of anaerobes). Plates were cultured for 24 h at 37 °C and 5% CO_2_, except for the Schaedler plate, which was cultured in an anaerobic chamber. The strains that grew were then identified by MALDI-TOF (Bruker Daltonics, Bremen, Germany) and a score of >2.2 was considered indicative of very reliable species identification. The microorganisms were freshly harvested before each use [41,42,43,44].

### 2.5. Bacterial Conversion of Urea to Ammonia and Analysis

Ammonia production was evaluated in standard medium (composed of peptone and yeast extract only) and sterilized MPAU (through a filter of 0.2 µm pore size) after inoculating both systems with *Providencia rettgeri*. *P. rettgeri* was precultured in 100 mL of standard medium for 24 h. Then, 1 mL from precultured standard medium was transferred to 100 mL of MPAU following the standard guidelines of CLSI and incubated at 37 °C with shaking at 120–130 rpm [45]. At each designated timepoint, including timepoint 0, approximately 10–15 mL of culture mixture was withdrawn using a sterile syringe and centrifuged at 900 rpm for 15 min to separate the urine scale. Then, approximately 2 mL of the supernatant was analyzed for OD_600_. The remaining supernatant was filtered through a syringe filter (0.2 µm) and analyzed for pH, ammonia and phosphate concentrations according to the protocols mentioned already for the enzymatic system (see Section 2.3). The samples used for the OD_600_ and pH were then combined and sterile filtered through a syringe filter (0.2 µm) back to the respective Erlenmeyer flasks to maintain a consistent reaction volume. The experiments were performed in triplicate and on three different occasions (*n* = 9). The quantification of citrate was carried out using orbitrap mass spectrometry employing a Thermo Scientific Q Exactive Orbitrap HR-MS (Darmstadt, Germany).

### 2.6. Analysis of Wood Ash and Its Extracts

The elemental composition of wood ash was determined using energy-dispersive X-ray spectroscopy (EDX), atomic absorption spectroscopy (AAS, AAnalyst 200, Perkin Elmer, Überlingen, Germany), triple-quadrupole inductively coupled plasma mass spectrometry (Agilent 8900 ICP-QQQ, Agilent Technologies, Santa Clara, CA, USA), and CHN analysis. The results of ICP-QQQ and CHN analysis have been presented as weight percent (wt %). EDX analysis was carried out at Leibniz Institute for New Materials in Saarbruecken using a Zeiss Sigma VP scanning electron microscope (Zeiss, Oberkochen, Germany) equipped with an Oxford Instruments X-Max EDX-unit with a 20 mm^2^ detection area. All measurements were carried out with 20 kV voltage and 30 µm aperture. The samples were measured in triplicate, and for each sample, three different areas were examined. CHN-Analysis was carried out using a Vario Micro Cube Analysator (Elementar Analysensysteme GmbH, Langenselbold, Germany). Thermogravimetric analysis was carried out using a TG 209F1 Iris (NETZSCH-Geraetebau GmbH, Selb, Germany) to estimate the CaO:CaCO_3_ ratio.

The ash was extracted with distilled water and also solubilized with different concentrations of HCl solution ranging from 0.1 M to 10 M. In general, 1 g of ash was suspended in 20 mL of solvent (either water or HCl) and heated to 100 °C for three hours. Upon cooling, the suspensions were filtered and the residue was washed with another 20 mL of respective solvent. The acidified extract and washing solutions were combined, resulting in 40 mL of the final solution, which was analyzed for Ca content by using AAS, whilst the insoluble fraction, subsequently referred to as acid-insoluble residue, was analyzed for its elemental composition by utilizing ICP-QQQ.

### 2.7. Reaction of Wood Ash Extract with Urea-Depleted MPAU and Characterization

The calcium-rich ash extracts were reacted with urea-depleted MPAU in a round-bottom flask and in a stoichiometric ratio to obtain a solid precipitate, which was filtered off and washed with distilled water. The powder was dried in an oven at 50 °C, weighed, and subsequently analyzed for the elemental composition by EDX, ICP-QQQ, and CHN analytical techniques. The leftover liquid of the reaction mixture and the fraction of water used for washing were combined, hereafter referred to as brine, and the combination was also evaluated using the above-mentioned techniques.

## 3. Results

**Figure 1:**
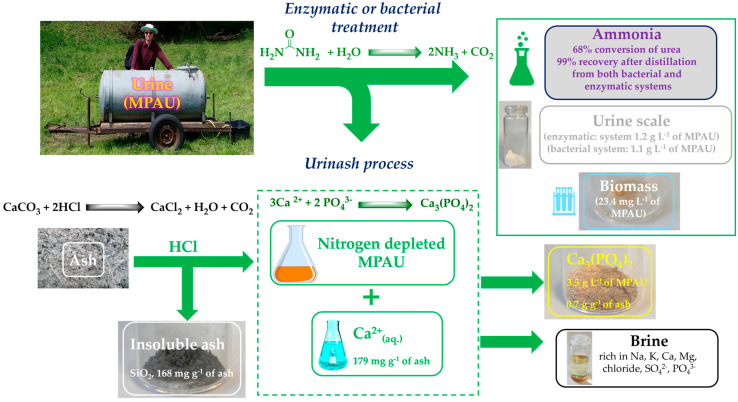
The urinash process can be employed to produce green NH_3_ and Ca_3_(PO_4_)_2_, as well as a few other interesting side products (urine scale, biomass, insoluble ash and brine). Photo credit Muhammad Jawad Nasim.

Taken together, our studies confirm that it is indeed possible to turn urine and ash into valuable new products, most notably green ammonia, in fair yields of 68% for both enzymatically or bacterially produced NH_3_ and calcium phosphate, and it is possible to almost quantitatively recover phosphate from urine and around 74% of the soluble calcium present in wood ash. The following sections will now present the urinash process and the individual products obtained during this sequential processing—as illustrated in Figure 1—in more detail.

### 3.1. Production of Ammonia Using Urease and Bacteria

**Figure 2:**
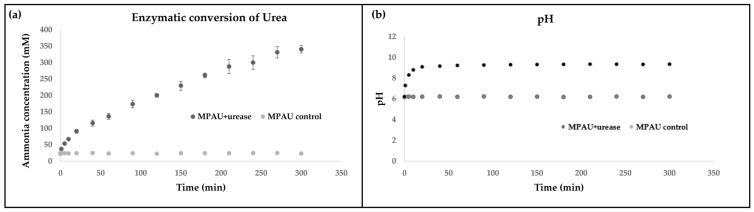
A time-dependent increase in the concentration of NH_3_ up to 340 mM (Panel (**a**)) and pH up to 9.6 (Panel (**b**)) was observed after the addition of urease (10 u mL^−1^) in MPAU. The experiments have been performed on three different occasions in triplicate (*n* = 9). Please note that the pH curve in Panel (**b**) levels off earlier compared to the concentration curve in Panel (**a**), as it is in essence logarithmic.

The first target compound in Figure 1 is green NH_3_. Bovine urine contains around 15 g urea per litre, in addition to 0.46 g ammonium, corresponding to molar concentrations of 250 mM and 26 mM, respectively [46,47]. Although urea itself is a valuable chemical, it is not easily possible to isolate it from urine, as also indicated already in Table 1. Thus, conversion of urea to NH_3_ combined with a subsequent distillation of the converted and naturally present NH_3_ at alkaline pH is one avenue to harvest—and indeed remove—nitrogen from urine. This can be achieved rather efficiently using the enzyme urease from jack beans, as shown in Figure 2. This conversion is fast and efficient, and yields of up to 68% are possible within a few hours of enzymatic incubation with urease (10 u mL^−1^), as illustrated in Figure 2a. As expected, this enzymatic conversion is accompanied by a sharp increase in pH from 6.6 to 9.6, also indicating that NH_3_ is formed and, thanks to its excellent solubility in water, mostly remains in solution (Figure 2b).

A nitrogen mass balance has also been performed to evaluate the efficiency of urea hydrolysis during urease treatment. Based on stoichiometry, 1 mol of urea produces 2 mol of ammoniacal nitrogen (NH_3_/NH_4_^+^), corresponding to a theoretical maximum of 500 mM NH_3_ (equivalent to 7 g N L^−1^) under the experimental conditions. The measured ammoniacal nitrogen concentration reached 340 mM (4.7 g N L^−1^), corresponding to a nitrogen recovery efficiency of approximately 68%. The deviation from the theoretical maximum can be attributed to ammonia volatilization, sampling and handling losses, and analytical uncertainty. This mass balance confirms substantial conversion of urea to plant-available nitrogen whilst also highlighting process losses under the conditions applied.

Although the use of urease represents a fast and easy method to produce NH_3_ from urea in urine, this enzyme is expensive and its production, usually from jack beans (*Canavalia ensiformis*), is not easily upscalable, at least not without considerable economic and ecological costs.

Another, perhaps more promising, avenue is therefore the use of microorganisms able to perform this conversion. In order to obtain the most suitable strain of bacteria, a real-life urine scale sample from a household urinal has been analyzed for relevant bacteria. Only a handful of organisms can thrive under these conditions and some are prone to producing ammonia. Indeed, the bacteria grown on the TSA–Sheep blood plate (non-selective) were identified as *Enterococcus faecalis* (*E. faecalis*, >10^4^ CFU mL^−1^) and *Streptococcus salivarius* (10^4^ CFU mL^−1^). The Gram-negative bacterial species grown on MacConkey agar were identified as *Paenalcaligenes suwonensis* (>10^4^ CFU mL^−1^) and *P. rettgeri* (~10^2^ CFU mL^−1^). The results of MALDI-TOF also revealed the presence of Gram-positive anaerobic *E. faecalis* (>10^4^ CFU mL^−1^) on CNA plate as well as in the Schaedler Agar.

**Figure 3:**
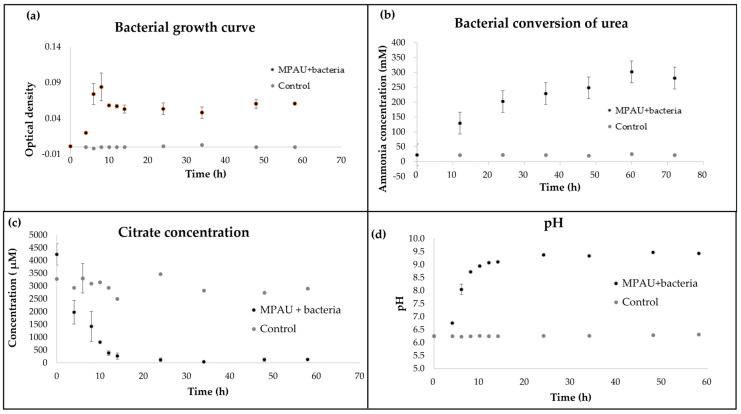
*P. rettgeri* are able to grow in MPAU (Panel (**a**)). A time-dependent increase in the concentration of NH_3_ has been observed after the addition of *P. rettgeri* in MPAU (Panel (**b**)). The citrate concentration decreases over time, reaching almost zero after 15 h, and its depletion may contribute to the stalling of the bacterial culture (Panel (**c**)). The increased amount of ammonia results in an increase in pH (Panel (**d**)). The experiments were performed in triplicate and on three different occasions (*n* = 9).

Amongst these organisms, *P. rettgeri* was identified as a urease-positive bacterium, which is well known to metabolize urea to NH_3_. It was subsequently cultured on MacConkey agar. As shown in Figure 3a, this microorganism also thrives in MPAU, reaches its maximum density after around 10 h, and converts around 60% of urea to ammonia in 60 h, as shown in Figure 3b. Notably, occasional sampling itself slightly decreases the yield, as uninterrupted fermentation for 72 h leads to an even higher yield of 68%. Since the pH of the solution, as expected, also increases during this time from 6.2 to 9.4, a much higher conversion yield is apparently not possible (Figure 3d). This may be achieved, however, by isolating and thus removing NH_3_ from the culture, which in turn lowers the pH to 6.2 again.

Notably, *P. rettgeri* also removes citrate from MPAU, most likely using it as one of its carbon sources, with the citrate concentration decreasing from initially 4.20 mM to just 0.26 mM after 14 h of incubation as quantified employing orbitrap mass spectrometry. This aspect of bacterial activity is also notable as it may remove some of the unwanted organic components of urine during fermentation (Figure 3c). It may also explain why bacterial growth stops after around ten hours, i.e., at around the same time the citrate fuel is depleted, although the bacteria then already present are apparently still active and able to convert urea to NH_3,_ as shown in Figure 3.

As mentioned already, NH_3_ does not escape the fermented MPAU spontaneously; thus, obtaining pure NH_3_ or ammonia solutions may require subsequent distillation or other ways of harvesting. This is indeed possible, and using a simple distillation setup, 63% of NH_3_ (based on initial urea content) can be recovered, albeit in dilute aqueous solutions. After removal of the ammonia, the pH value of the remaining solution returns to around 6.2, and further bacterial fermentations with a fresh bacterial culture are then possible again.

### 3.2. Urine Scale and Biomass

During the fermentation process, a time-dependent increase in the amount of a solid precipitate was observed, which is referred to here as “urine scale”. After the bacterial conversion had ceased, the whole mixture was therefore centrifuged initially at 900 rpm to harvest urine scale and then at 7000 rpm to isolate biomass. In total, 1.2 g L^−1^ and 2.2 g L^−1^ of urine scale were obtained from MPAU in the enzymatic and bacterial systems, respectively. ICP-QQQ analysis confirmed the presence of mostly inorganic elements, such as P (16.0%), Mg (10.4%), Ca (4.2%), Na (2.9%) and K (2.2%), whilst CHN analysis further showed C, H, and N contents of 0.7%, 5.0%, and 4.2%, respectively. These quantitative data align well with the composition of struvite (MgNH_4_PO_4_·6H_2_O), which contains O (65.2%), P (12.6%), Mg (9.9%), H (6.6%) and N (5.7%). The deviations in the values can be explained by co-precipitation with about 10% of calcium phosphate. The elemental analysis via EDX further re-affirmed the presence of P, Ca, Mg, Na and K with traces of S. Intriguingly, the composition of urine scale isolated from bacterial and enzymatic sources was more or less identical.

Besides the urine scale, around 23 mg L^−1^ dry weight of the biomass was obtained from a typical 100 mL *P. rettgeri* culture. CHN analysis has affirmed that this biomass consists of 42.7% C, 6.6% H and 12.6% N, with EDX also pointing to the presence of additional elements such as O, P, S, K and Cl.

The main leftover of this enzymatic process, the nitrogen-depleted MPAU, is still rich in phosphate, and thus, the next step in this waste-to-value avenue involves phosphate recovery, this time with a chemical rather than enzymatic or bacterial reaction and the use of calcium ions as precipitant obtained from ash.

### 3.3. Wood Ash and Acid Digestion

To follow this avenue, the wood ash from the thermal station of St. Ingbert was analyzed for some of its key components, especially calcium, using EDX, ICP-QQQ, CHN-S and AAS. ICP-QQQ revealed the presence of Ca (17.6%), K (26.4%), S (8.6%), Mg (3.9%), Fe (3.4%), Al (2.8%), and traces of P, Si, and Mn.

Indeed, Ca is of special interest in the urinash process, yet for this purpose, it must be solubilized first. Using pH measurements and also thermogravimetric studies, it seems that roughly 80% of the total Ca is present as sparingly soluble carbonate and only around 20% as the more soluble, and thus potentially phosphate-reactive, oxide/hydroxide, as shown in Figure S2 in the SI. Thus, reacting Ca in ash with a phosphate solution directly may be inefficient.

Wood ash has therefore been extracted with distilled water directly, and has also been reacted with different concentrations of HCl ranging from 0.1 M to 10 M. As expected, the Ca concentration in the supernatant increases more than ten-fold from just 15.3 mg per gram of ash used in the aqueous extract to 179 mg after HCl (5 M) treatment. Similarly, a lower amount of insoluble ash (168 mg from 1 g ash) was obtained when extractions were carried out with 5 M HCl compared to aqueous extraction (620 mg of insoluble residue per gram of ash). The acid-insoluble residues from ash were analyzed for their chemical composition employing both EDX and ICP-QQQ and contained mostly silicon, pointing towards a silicate-like material (similar to quartz sand), along with smaller amounts of Ba, K, Ca and S. Besides Si, the acid-insoluble residue also contained K, Ca, S, Al, Na, C and Mg, as observed in the EDX spectrum. The supernatant of ash treated with 5 M HCl and containing 112 mM concentrations of Ca^2+^ ions was therefore deemed appropriate for further reactions.

### 3.4. Precipitation of Calcium Phosphate from Nitrogen-Depleted Urine and Acid-Solubilized Ash

This acidic supernatant was therefore employed in the true culmination of the urinash process, namely the reaction of phosphate from nitrogen-depleted urine (NH_3_ has been distilled off) and acid-solubilized wood ash, i.e., the two leftovers described in Section 3.1 and Section 3.3, respectively. Indeed, once this reaction is carried out under a stoichiometry of Ca and phosphate of 3 to 2, and at an amenable pH of around 8.2 (adjusted using an excessive amount of NaOH), a yellow precipitate is obtained instantaneously and in a quantitative yield of around 99% with regard to Ca and phosphate. As for the precipitation reaction, mixing 1000 mL of N-depleted urine containing 1.23 g of PO_4_^3−^ with 200 mL of ash extract containing 0.65 g of Ca^2+^ produced approximately 3.5 g of calcium phosphate precipitate, corresponding to the consumption of about 99% of both phosphate and calcium from the supernatant, which only contained 0.5% of the initial phosphate and 0.03% of the initial calcium after the reaction.

According to ICP-QQQ analysis, the precipitate itself consists of 18.6% Ca, 11.2% P, and 7.1% Na, along with trace amounts of Mg, Fe, Mn, and Al, which may be responsible for the light-brown colour of the substance. The solid is therefore most likely a mixture of different calcium phosphates with varying stoichiometries, including amorphous calcium phosphate, Brushite (CaHPO_4_·2H_2_O), and Hydroxyapatite (Ca_10_(PO_4_)_6_(OH)_2_), potentially with minor contributions of sodium- and magnesium-substituted calcium phosphates. EDX data further affirms the presence of Ca, P and Mn. The pigmentation may originate from the presence of Mn in this precipitate, as affirmed by both ICP-QQQ and EDX analysis.

As for the total yield and efficiency of the urinash process, and considering the initial amounts of PO_4_^3−^ in MPAU and Ca in wood ash, 54% of the PO_4_^3−^ initially present in the MPAU and approximately 74% of the Ca originally present in the ash were recovered successfully in the calcium phosphate material. The remaining PO_4_^3−^ initially present in MPAU was found in the urine scale, adding up to almost 99% of phosphate recovery from MPAU and virtually no phosphate waste in the urinash process.

### 3.5. Leftovers

After harvesting the desired calcium phosphate product and separating it from the supernatant via filtration, the only leftover of the urinash process, besides the acid-insoluble residue of the ash, was a salty brine rich in the soluble minerals from both the initial MPAU and the soluble fraction of the wood ash. Since the precipitation of phosphate with calcium is almost quantitative at pH 8.2, phosphate had been removed from the solution more or less completely and thus could not be detected in the brine, whilst nitrogen, as measured by CHN analysis, was just 4.1%.

According to EDX and ICP-QQQ analysis, and in accordance with the initial analysis of wood ash (by EXD and ICP-QQQ) described in Section 3.3, brine has only traces of P and Ca. Brine primarily comprises of minerals, including mostly alkaline metal ions, such as Na (1114 mM) and K (58 mM). Intriguingly, S (22 mM) was also found in the brine. The presence of such a large amount of Na may originate from the neutralization of the acidified ash extract before its reaction with N-depleted urine.

Thus, besides a few alkaline metal ions, such as K, some sulfate, etc., the brine is more or less devoid of any valuable or indeed toxic ions and, as the only waste material, may be processed further or discarded.

Together with the other products harvested along the process, these leftovers and their potential uses will now be discussed in more detail.

## 4. Discussion

Taken together, the data obtained in this initial feasibility study demonstrate that it is indeed possible to convert a combination of two environmentally damaging waste products, namely (mammalian) urine and wood ash, into a set of valuable materials, including ammonia and calcium phosphate, in good purity and excellent yields, and to simultaneously reduce the content of environmentally damaging nitrogen and phosphate. Along the way, the valuable target products are joined by a few more or less harmless and easy-to-handle side products, such as bacterial biomass, inorganic urine scale, insoluble “sandy” fractions of ash rich in silicate, and a single “salty” brine fraction containing Na, K, Cl and S—which, together with the main products, may be of interest and processed further.

### 4.1. Green Ammonia

The production of “green” NH_3_ from urine can be achieved via enzymatic or bacterial conversion. Considering the speed of the conversion and yields, both approaches provide more or less similar outcomes, yet the enzymatic system requires a constant supply of a rather expensive enzyme, which so far is usually extracted in small(er) amounts from jack beans and soybeans [48,49,50,51]. Upscaling the production of this enzyme to cope with millions of litres of urine may not be feasible. The bacterial conversion, on the other hand, is considerably slower and also may require several fermentation–distillation cycles, as the bacteria cannot carry out a full conversion in one go, most likely because of the formation of NH_3_ and/or increases in pH. Such problems are common, as the alcoholic fermentation with yeast shows, where ethanol production also ceases at around 10 to 20% despite the remaining sugars [52]. It is also possible that bacterial growth of *P. rettgeri* ceases after around 12 h of incubation because at around the same time citrate is used up, although the bacteria seem to remain viable as NH_3_ production continues for at least another 48 h. Thus, the use of *P. rettgeri* seems to be the most attractive avenue, at least today.

Still, *P. rettgeri* is an opportunistic pathogen and therefore requires appropriate biosafety handling under controlled laboratory conditions. Its use in engineering or environmental applications would necessitate strict risk assessment and containment measures. For future applications, the use of non-pathogenic urease-positive strains or engineered microbial systems is recommended to ensure biosafety while maintaining process functionality. Such a bacterial fermentation process may indeed be carried out on a larger scale and may also be optimized, considering that real urine is also more complex than MPAU and may contain traces of other materials, chemicals and even antibiotics, issues that are worth addressing in a more technical follow-on study (see also Section 4.3 on limitations of this study). A follow-on study may also consider alternative avenues to collecting NH_3_ from the solution, as distillation, despite being the initial method of choice, is simple yet also energy-intensive and, in any case, only results in ammonia solutions and not in pure NH_3_. The use of cold traps and low(er) temperatures may be attractive, as may be the use of a vacuum.

Ammonia is perhaps not the most valuable chemical, yet producing it naturally as “green ammonia” and not via the traditional Haber–Bosch process has its attraction, as this gas is used increasingly in fuel cells [53]. Thanks to its comparably high boiling point of −33.4 °C, and thus comparable ease of handling, NH_3_ is a worthy carbon-free fuel and possible alternative in addition to H_2_, probably the only high-energy carbon-free fuel one may envisage, as other non-carbon alternatives, such as hydrazine (N_2_H_4_) or H_2_S, are indeed highly problematic and entirely unsuitable for large-scale fuel applications [54,55]. A rough estimate indicates that around 0.4 million t of “green ammonia” could be produced from bovine urine alone in Germany each year, assuming all of the bovine urine is being collected and processed, compared to three million t produced by traditional methods [56]. Clearly, such a collection of urine from farm animals and perhaps also humans may be challenging today, yet poses a hurdle that can be overcome, similar to the collection of paper, glass and plastics.

### 4.2. Calcium Phosphate

Beyond ammonia, the most valuable product of this process is calcium phosphate, mostly in the form of apatite, which is of considerable importance in agriculture and industry, for instance as a fertilizer. Based on our initial results, it is possible to recover the phosphate present in MPAU in an almost quantitative yield, as urine scale and, most efficiently, via precipitation with a stoichiometric amount of calcium ions. Notably, the product formed in a mixture of different calcium phosphate salts, which is not surprising, as there are at least three different calcium (hydrogen) phosphate salts possible.

As for the harvesting of calcium from ash, it is apparent that wood ash formed under standard conditions contains mostly carbonates and thus calcination at elevated temperatures of around 1050 °C or an acid digest at 100 °C are necessary in order to liberate the calcium ions needed for the calcium phosphate reaction [57]. Indeed, the concentration of calcium in solution increases tenfold once 5 M HCl is applied as solvent compared to H_2_O. The need for HCl and its various implications are discussed below.

As for calcium phosphate itself, such salts have been mined traditionally in countries such as the Republic of Nauru and the Republic of Kiribati, where this mining activity has left a trail of devastation [58,59]. Not surprisingly, these mining activities have now been mostly outlawed and new, more sustainable sources are being sought. In many countries, the focus is indeed on sewage, where some of the more promising methods of harvesting phosphate from these diluted waste waters involve precipitation as Ca or Mg salts, and wood ash and fly ash have already been considered as sources of such metal ions (see also Table 1) [60,61,62,63].

**Figure 4:**
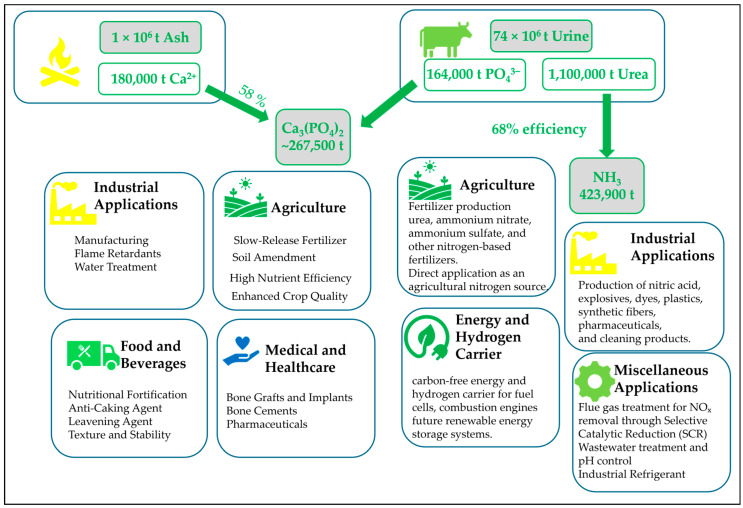
The amounts of bovine urine and wood ash produced each year in Germany could theoretically add up to 267,500 t of calcium phosphate (Ca_3_(PO_4_)_2_) via the urinash process, assuming all of bovine urine and 58% of wood ash are collected and the reaction proceeds completely. The applications of Ca_3_(PO_4_)_2_ are not limited to agriculture but also encompass medical, food, and industrial applications.

The urinash strategy follows these leads and, by liberating the Ca from wood ash by using HCl, achieves yields which may be suitable for large(r) scale manufacture. Assuming all of the phosphate-rich cattle urine could be collected together with 58% of wood ash produced in Germany , this would add up to a theoretical total of 267,500 t of green Ca_3_(PO_4_)_2_ each year (Figure 4). In comparison, Germany currently consumes approximately 480,000–520,000 t of phosphate fertilizer (expressed as P_2_O_5_ equivalent) annually, corresponding to roughly 1.0–1.2 million t of Ca_3_(PO_4_)_2_ equivalents. Assuming an annual production potential of 267,500 t Ca_3_(PO_4_)_2_ equivalent from the urinash process, the recovered phosphate could theoretically cover approximately 22–27% of Germany’s current calcium phosphate demand.

### 4.3. Limitations of the Study

As with most feasibility studies, the urinash process also leaves some questions open and still has quite a few limitations, especially when it comes to transferring this strategy and the initial data from a laboratory setting to a real-life scenario. These limitations include, for instance, the differences between MPAU and real urine, the collection and handling of wood-derived products, toxic metal ions in ash, and issues surrounding large-scale digestion of wood ash with acids or other (corrosive) chemicals—which obviously also liberates considerable amounts of CO_2_. Eventually, the energy balance and economic input for output of the process as a cornerstone of a techno-economic assessment also needs to be considered when moving from the laboratory to an industrial scale. As these limitations are important and may also stimulate further research, they are presented here in more detail.

First and foremost, MPAU has been employed in this study to provide a controlled and reproducible experimental matrix. Although MPAU closely resembles the major constituents of urine, real human and animal urine samples generally comprise a far more complex mixture of organic compounds, suspended solids, pharmaceuticals, including antibiotics, as well as naturally occurring microbial communities and possibly some toxic metal ions. These components may interfere with enzymatic, microbial or chemical processes, such as urea hydrolysis, microbial activity, and the efficiency of NH_3_ recovery. Nevertheless, since urea is the dominant nitrogen-containing compound in urine, the fundamental recovery mechanisms investigated here are expected to remain relevant under practical conditions. Validation using real urine streams will be an important next step to assess the robustness of the process under more realistic operating conditions.

Similarly, the use of wood ash as a calcium source also raises several concerns. The long-term sustainability of this approach relies primarily not only on the availability of suitable biomass residues but also on compliance with relevant sustainability and certification requirements, which are outside the scope of the present study. Furthermore, wood ash may contain trace amounts of metals such as Zn, Cu, Pb, Cd, Cr, and Ni, depending on the biomass source and combustion conditions. During acid extraction and subsequent Ca_3_(PO_4_)_2_ precipitation, some of these elements may be transferred to the recovered products. Since heavy metal concentrations have not been determined in this study, at least not in detail, the environmental safety and agricultural suitability of the recovered Ca_3_(PO_4_)_2_ cannot yet be fully assessed. Future work should therefore include detailed elemental analyses of the ash feedstock and the recovered products.

To determine the maximum recoverable Ca content, wood ash has been digested using 5 M HCl. While this approach is suitable for laboratory-scale characterization, the use of concentrated HCl may not be practical at industrial scale due to reagent consumption, corrosion issues, and waste treatment requirements. Further optimization should focus on reducing acid demand, implementing acid recycling strategies, or exploring alternative extraction methods. A particularly interesting possibility is the integration of chlor–alkali electrolysis, whereby the brine generated during the process could be converted into reusable HCl and NaOH using renewable electricity and a fuel cell converting H_2_ and Cl_2_ to HCl. Although such concepts remain speculative, they illustrate potential pathways towards a more circular process design.

Besides NH_3_ and Ca_3_(PO_4_)_2_, the byproducts may also offer opportunities for additional resource recovery. The microbial biomass, for instance, could potentially be reused as inoculum or processed for the recovery of proteins, amino acids, or other valuable organic compounds. Similarly, the acid-insoluble silicate fraction remaining after ash digestion may serve as a source for secondary minerals. In contrast, the brine produced by the process currently represents the main residual stream and requires further characterization before suitable reuse options can be identified.

Eventually, the experiments presented here were performed under batch conditions. While this allowed a detailed investigation of individual process steps, industrial implementation would most likely rely on continuous operation using interconnected reactors for urea hydrolysis, biological conversion, and Ca_3_(PO_4_)_2_ precipitation. Such systems introduce additional challenges related to process control; mass transfer; the handling of solids, liquids, and even gases; and operational stability.

Similarly, the economic and environmental performance of the process will ultimately depend on factors such as reagent recycling, energy consumption, and product valorisation. A comprehensive techno-economic and life-cycle assessment, coupled with pilot-scale testing, will therefore be necessary before the full potential of the urinash process can be evaluated. Although it still suffers from volatilization of some NH_3_ and incomplete hydrolysis, the process has low operational costs and moderate energy requirements, with moderate to high scalability. As far as one may judge in light of the data available already, its main advantages, despite its limitations, therefore dominate and include simple operation, low chemical input, removal of waste materials and suitability for decentralized circular economy applications, possibly in rural areas.

## 5. Conclusions

As a contribution to ongoing research in the field of circular processes and sustainable materials, and in light of the high demand for sustainable sources for ammonia and fertilizers, this study has confirmed that it is possible to use a combination of urine and wood ash to produce such important substances, in good quality and yield. The ensuing urinash process is based on several biological and chemical conversions which together ensure maximum use of the waste, good yields and purity of products, and minimal amounts of byproducts and leftovers. The latter are unavoidable, of course, considering the waste materials at hand, and may be explored further as they may also still contain some valuable ingredients.

In the future, it is now necessary to optimize these conversions, to explore larger-scale handling of processes and materials, and also to consider logistical, economic, and ecological factors. As mentioned already in the Discussion, collecting and handling urine is perhaps the most challenging issue, yet this may need to be overcome, if one is serious about a sustainable economy, in a similar manner to how paper, glass or plastic is collected these days. Obtaining wood ash is more straightforward, although there may be issues related to heavy metals—these may collect in the brine, together with other soluble inorganic and organic substances that need to be considered in more detail.

In any case, the urinash strategy described here has considerable potential to minimize the impact of nitrogen and phosphorous on the environment and, at the same time, can provide industry, agriculture and other users with raw materials desperately needed in times of global turmoil, produced often locally and from highly sustainable sources.

## Figures and Tables

**Table 1:** Comparison of existing urine upcycling technologies in terms of efficiency, cost and scalability.

Technology	Main Recovered Products	Recovery Efficiency	(a): Operational Cost (b): Energy (c): Requirement Scalability	Advantages	Limitations
**Struvite precipitation [25,26]**	Struvite fertilizer (MgNH_4_PO_4_·6H_2_O)	High P recovery; limited N recovery	(a): Moderate (b): Low to moderate (c): High	Mature and widely studied technology; P recovery	Requires addition of Mg and pH control; incomplete N recovery
**Ammonia stripping** **[27,28]**	NH_3_/(NH_3_)_2_SO_4_	High N recovery (>80%)	(a): Moderate to high (b): High (c): High	Efficient N recovery; industrial applicability	High energy demand and alkaline conditions required
**Membrane separation (RO, FO, membrane distillation)** **[29,30]**	Concentrated mineral solution	High mineral recovery	(a): High (b): Moderate to (c): high Moderate	High separation efficiency and water recovery	Membrane fouling and high capital costs
**Electrochemical recovery** **[31,32]**	NH_3_ or mineral concentrates	Moderate to high	(a): High (b): High (c): Moderate	Reduced chemical consumption; selective recovery possible	Energy intensive;costs for electrodes
**Biological nitrification/denitrification** **[33,34]**	Stabilized N- compounds	Variable	(a): Moderate (b): Moderate (c): High	Environmentally friendly and biologically driven	Requires careful process control

## Data Availability

Raw data supporting the conclusions of this article will be made available by the authors on request.

## Supplementary Materials

The following supporting information can be downloaded at: https://www.mdpi.com/article/10.3390/bioengineering13070720/s1. Figure S1. Thermogravometric analysis (TGA) for Ash, CaO, CaCO_3_ as well as different combinations of CaO, Ca-CO_3_ and Carbon; Figure S2. EDX spectrum of ash; Figure S3. EDX spectra of urine scale obtained from the enzymatic (a) and bacterial (b) systems; Figure S4. EDX spectrum of biomass; Figure S5. EDX spectrum of the precipitate obtained from the ammonia depleted MPAU; Figure S6. EDX spectrum of dried residues from the leftover brine. Table S1. Composition of multipurpose artificial urine (MPAU).
