# Supplementary material for: From Waste to Value: Urine and Ash as Sustainable Sources for Green Ammonia and Calcium Phosphate Fertilizers
Source: Bioengineering (Basel). 2026 Jun 24;13(7):720. doi: 10.3390/bioengineering13070720 (PMC13404784; doi:10.3390/bioengineering13070720)
Supplement: Supplementary file 1 [file bioengineering-13-00720-s001.zip › bioengineering-4346517-supplementary.pdf]

bioengineering

MDPI

Article

From waste to value: Urine and ash as sustainable sources for green ammonia and calcium phosphate fertilizers

Zhengyu Li, Eduard Tiganescu, Kevin Böhm, Muhammad Jawad Nasim * and Claus Jacob *

Division of Bioorganic Chemistry, School of Pharmacy, Saarland University, 66123 Saarbruecken, Germany; zhli00012@stud.uni-saarland.de (Z.L.); s9edtiga@stud.uni-saarland.de (E.T.); kevin.boehm@uni-saarland.de (K.B.)

* Correspondence: jawad.nasim@uni-saarland.de (M.J.N.); c.jacob@mx.uni-saarland.de (C.J.); Tel.: +49-681-302-57335 (M.J.N.); +49-681-302-3129 (C.J.)

Table S1. Composition of multipurpose artificial urine (MPAU)

Reagent No.	Formula	Name	conc. in MPAU (g L^{-1})	conc. In MPAU (mM)
1	Na_2SO_4	sodium sulfate	1.7	12.0
2	$Na_3C_6H_4O_7$	tri-sodium citrate	0.7	2.8
3	$C_4H_7N_3O$	creatinine	0.8	7.2
4	CH_4N_2O	urea	15.0	250.0
5	KCl	potassium chloride	2.3	31.0
6	NaCl	sodium chloride	1.8	30.0
7	$CaCl_2{\cdot}2H_2O$	calcium chloride	0.2	1.7
8	NH_4Cl	ammonium chloride	1.3	23.7
9	$K_2C_2O_4{\cdot}H_2O$	potassium oxalate	0.02	0.1
10	$MgSO_4{\cdot}7H_2O$	magnesium sulfate	1.1	4.4
11	NaH_2PO_4	sodium dihydrogen phosphate	2.2	18.7
12	Na_2HPO_4	sodium phosphate monobasic	0.7	4.7

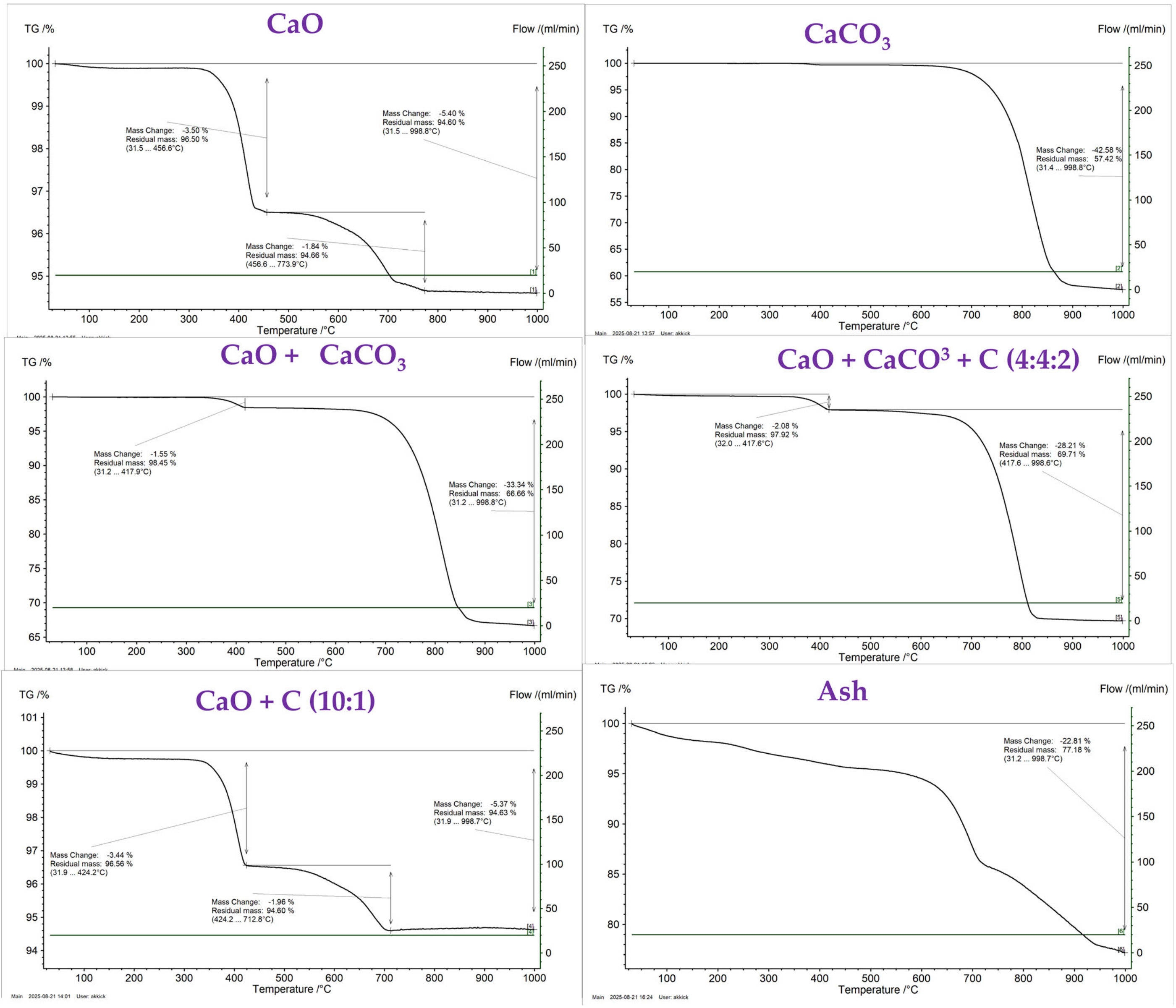

Figure S1. Thermogravometric analysis (TGA) for Ash, CaO, $CaCO_3$ as well as different combinations of CaO, $CaCO_3$ and Carbon

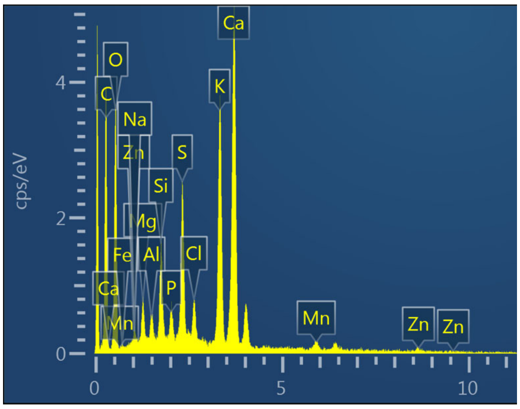

Figure S2. EDX spectrum of ash

https://doi.org/10.3390/bioengineering13070720

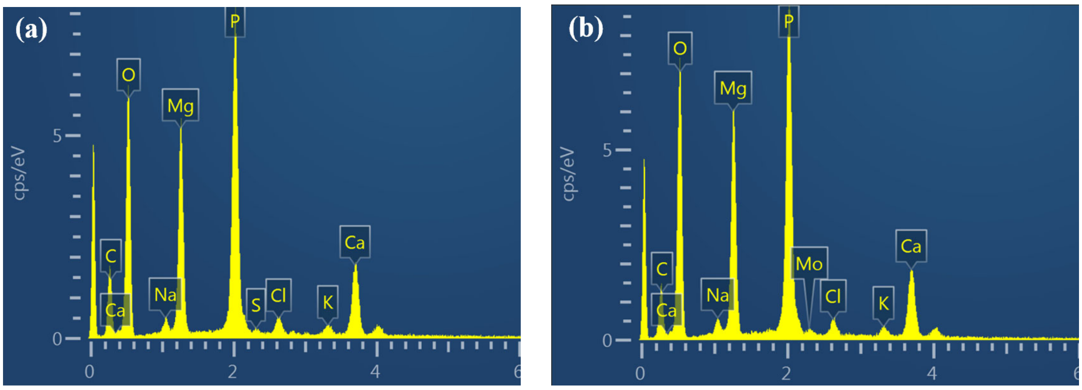

Figure S3. EDX spectra of urine scale obtained from the enzymatic (a) and bacterial (b) systems

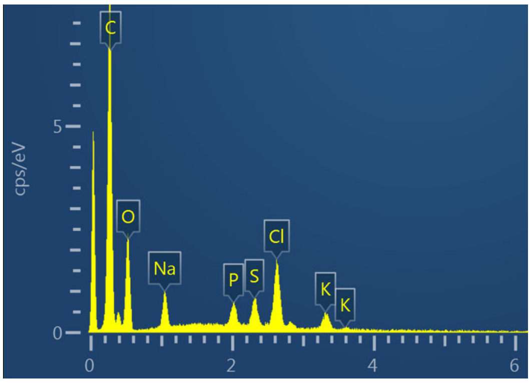

Figure S4. EDX spectrum of biomass

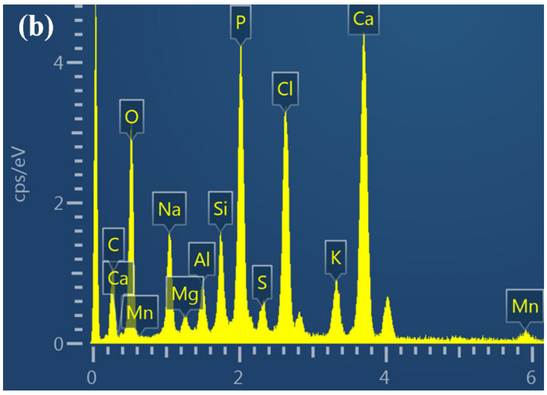

Figure S5. EDX spectrum of the precipitate obtained from the ammonia depleted MPAU

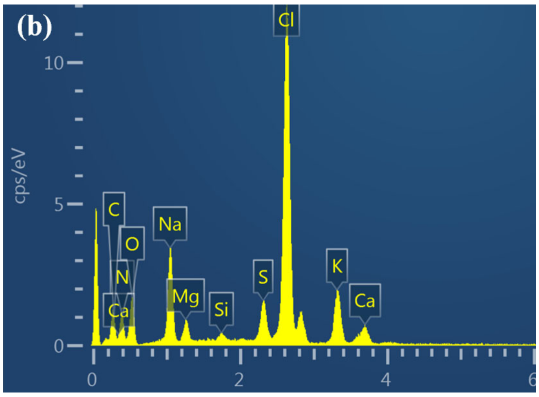

Figure S6. EDX spectrum of dried residues from the leftover brine

Disclaimer/Publisher's Note: The statements, opinions and data contained in all publications are solely those of the individual author(s) and contributor(s) and not of MDPI and/or the editor(s). MDPI and/or the editor(s) disclaim responsibility for any injury to people or property resulting from any ideas, methods, instructions or products referred to in the content.

https://doi.org/10.3390/bioengineering13070720
